# Supplementary material for: Visualization and validation of twin nucleation and early-stage growth in magnesium
Source: Nat Commun. 2022 Jan 10;13:20. doi: 10.1038/s41467-021-27591-z (PMC8748725; doi:10.1038/s41467-021-27591-z)
Supplement: Supplementary file 1 — Supplementary Information [file 41467_2021_27591_MOESM1_ESM.pdf]

Supplementary Information

Visualization and Validation of Twin Nucleation and Early-stage Growth in Magnesium

Author List: Lin Jiang [1,2], Mingyu Gong [3], Jian Wang [4], Zhiliang Pan [1], Xin Wang [1], Dalong Zhang [1], Y. Morris Wang [5], Jim Ciston [6], Andrew M. Minor [6,7], Mingjie Xu [1], Xiaoqing Pan [1,8], Timothy J. Rupert [1], Subhash Mahajan [9], Enrique J. Lavernia [10], Irene J. Beyerlein [11], Julie M. Schoenung [1*]

Affiliations:

[1] Department of Materials Science and Engineering, University of California, Irvine, CA 92697, USA

[2] Materials & Structural Analysis Division, Thermo Fisher Scientific, Hillsboro, OR 97124, USA

[3] State Key Lab of Metal Matrix Composites, Shanghai Jiao Tong University, Shanghai 200240, China

[4] Department of Mechanical & Materials Engineering, University of Nebraska-Lincoln, Lincoln, NE 68588, USA

[5] Department of Materials Science and Engineering, University of California, Los Angeles, Los Angeles, CA 90095, USA

[6] Molecular Foundry, Lawrence Berkeley National Laboratory, CA 94701, USA

[7] Department of Materials Science and Engineering, University of California, Berkeley, CA 94720, USA

[8] Department of Physics and Astronomy, University of California, Irvine, CA 92697, USA

[9] Department of Materials Science and Engineering, University of California, Davis, CA 95616, USA

[10] National Academy of Engineering, Irvine, CA 92617, USA

[11] Department of Mechanical Engineering and Materials, University of California, Santa Barbara, CA 93101, USA

* Corresponding author: Julie M. Schoenung <schoenuj@uci.edu>

Supplementary Figures

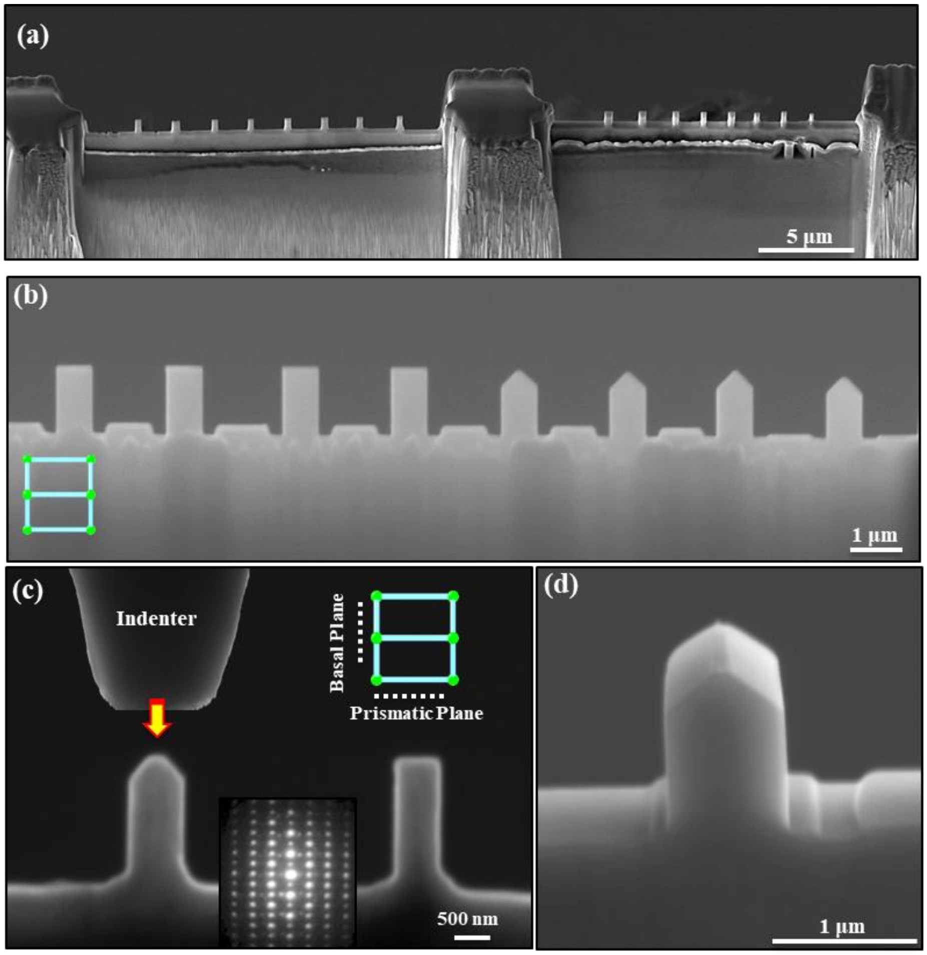

Supplementary Figure 1. Manipulating twinning via design of pillar geometry: (a) SEM image showing H-Bar type foils with the pillars; (b) SEM image showing the pillars on the H-Bars with different geometries; (c) SEM image showing the in-situ compression set-up. The inset is a selected area diffraction pattern revealing the orientation of the pillar. (d) SEM image showing the three-dimensional features of the pillars with 750 nm thickness.

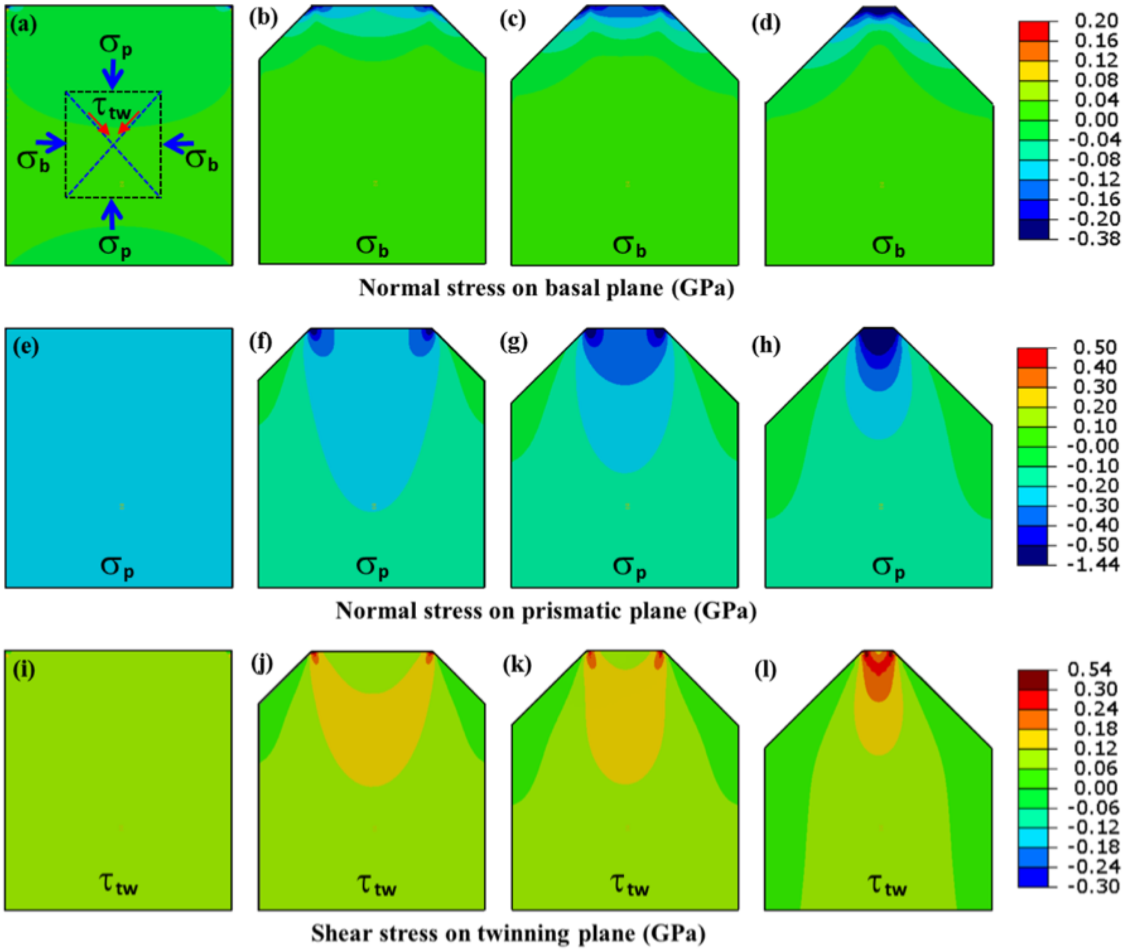

Supplementary Figure 2. Simulated stress distributions inside the pillars with different tip sizes. Simulated normal stress on the basal plane (σ_b) inside the pillars with tip size of: (a) 750 nm, (b) 400 nm, (c) 250 nm, and (d) 100 nm. Simulated normal stress on basal plane (σ_p) inside the pillars with tip size of: (e) 750 nm, (f) 400 nm, (g) 250 nm, and (h) 100 nm. Simulated normal stress on the $(10\bar{1}2)$ twinning plane (τ_{tw}) inside the pillars with tip size of: (i) 750 nm, (j) 400 nm, (k) 250 nm, and (l) 100 nm. The compression on the prismatic plane is much higher than the resolved shear stress on the twin planes. Stress/strain concentrators are located at the corners of the pillars.

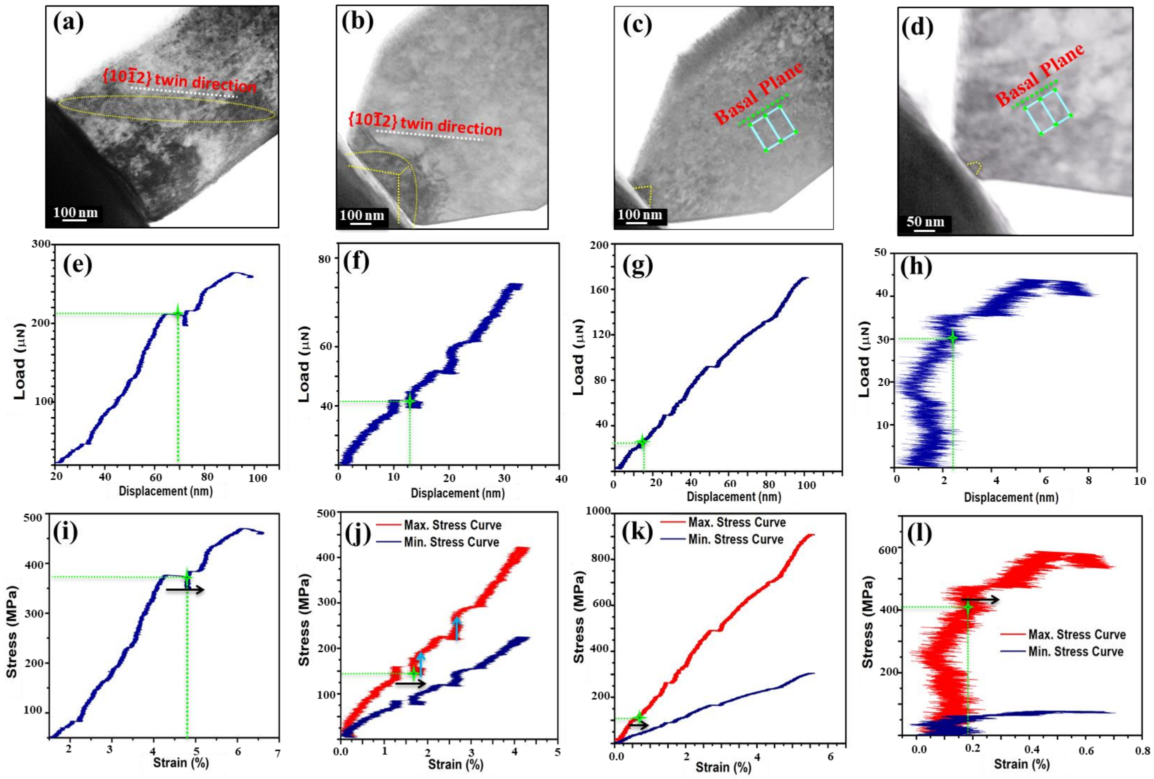

Supplementary Figure 3. TEM images and related in-situ compression curves for the single-crystal pillars with various geometries. The TEM snapshots correlate to the moment when the twin nucleated in: (a) a conventional pillar with 750 nm width, and a truncated wedge-shaped pillar with top width of: (b) 400 nm, (c) 250 nm, and (d) 100 nm. The related in-situ load/displacement curves of: (e) the conventional pillar, and the truncated wedge-shaped pillar with a top width of: (f) 400 nm, (g) 250 nm, and (h) 100 nm. The related in-situ stress curves of: (i) the conventional pillar and the wedge-shaped pillar with top width of: (j) 400 nm, (k) 250 nm, and (l) 100 nm. The max. stress curve was calculated as the ratio of compression loading relative to the cross section of the pillar top. The min. stress curve was calculated as the ratio of compression loading relative to the cross section of the pillar bottom. The horizonal arrows in the compression curves indicate strain burst events associated with twinning deformation. The vertical arrows in the (j) indicate stress burst events associated with twinning deformation. The star in the compression curves indicate the point where the twin nucleus can first be identified.

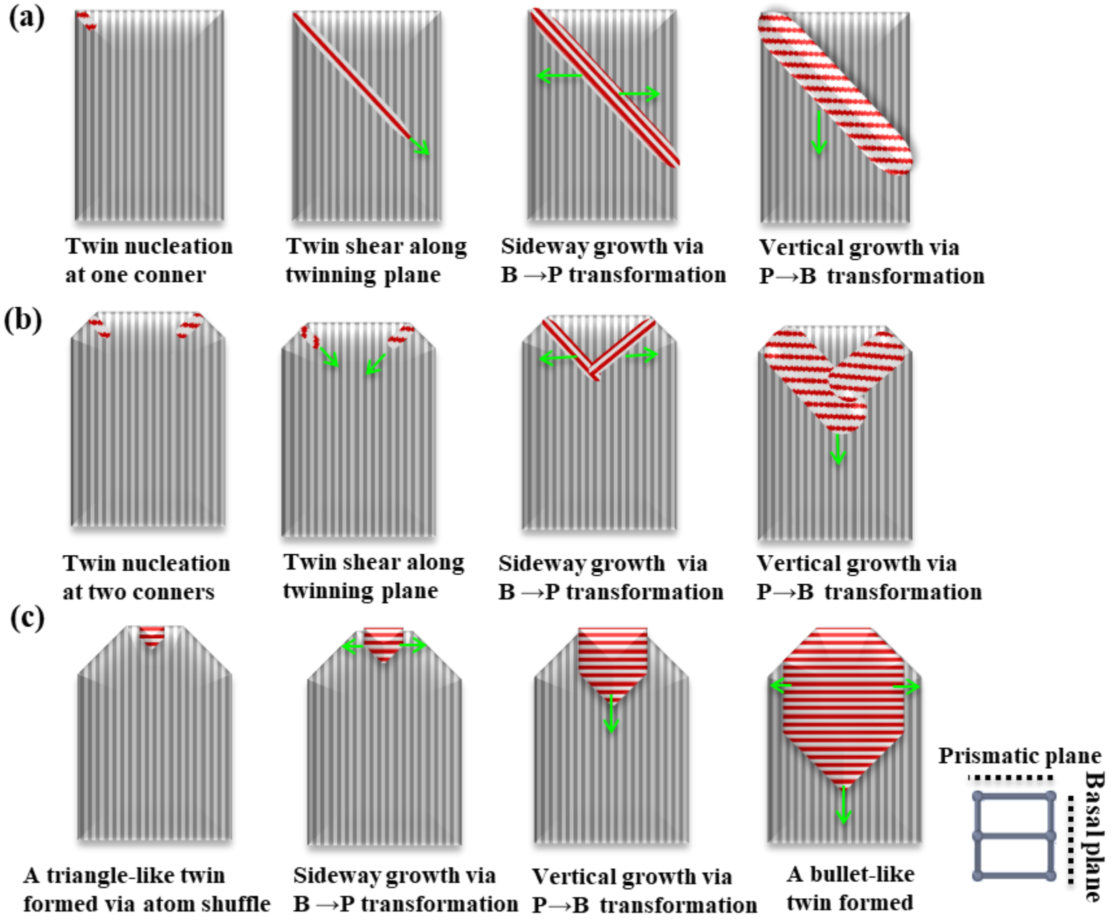

Supplementary Figure 4. The summary of sample-shape dependent twinning behavior in deformed Mg. (a) Schematic of twin nucleation and early-stage growth in conventional pillars. (b) Schematic of twin nucleation and early-stage growth in the truncated wedge-shaped pillars with 400 nm top. (c) Schematic of twin nucleation in the truncated wedge-shaped pillars with 250 nm and 100 nm top. The schematics were drawn based on the in-situ compression results.

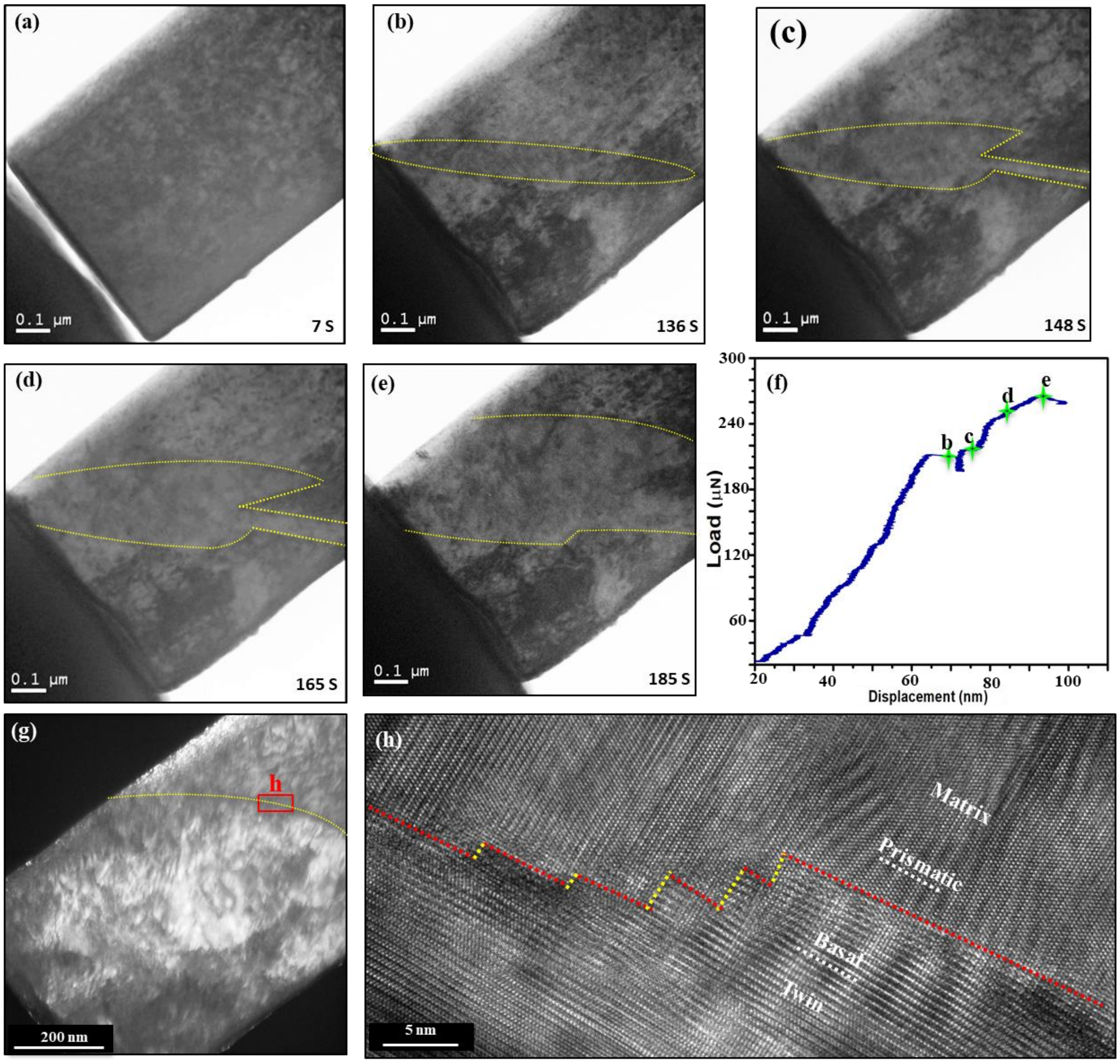

Supplementary Figure 5. Dynamic twinning captured in a conventional Mg pillar. (a-e) TEM snapshots showing microstructure evolution, (f) the in-situ compression loading-displacement curve. The dashed lines indicate the twin boundaries. The twin propagates along the shear plane without apparent shear offset on the surface (b) and then thickens along the basal plane (c-e). The point b in (f) correlates to the moment when the twin first nucleated. The remarkable strain burst (with a magnitude of approximately 5 nm) is due to twinning. (g-h) Post-mortem TEM images revealing the orientation relationship between matrix and twin. (h) HRTEM image of the red rectangle region in (g). The yellow dashed lines indicate basal/prismatic interfaces and red dashed lines indicate prismatic/basal interfaces.

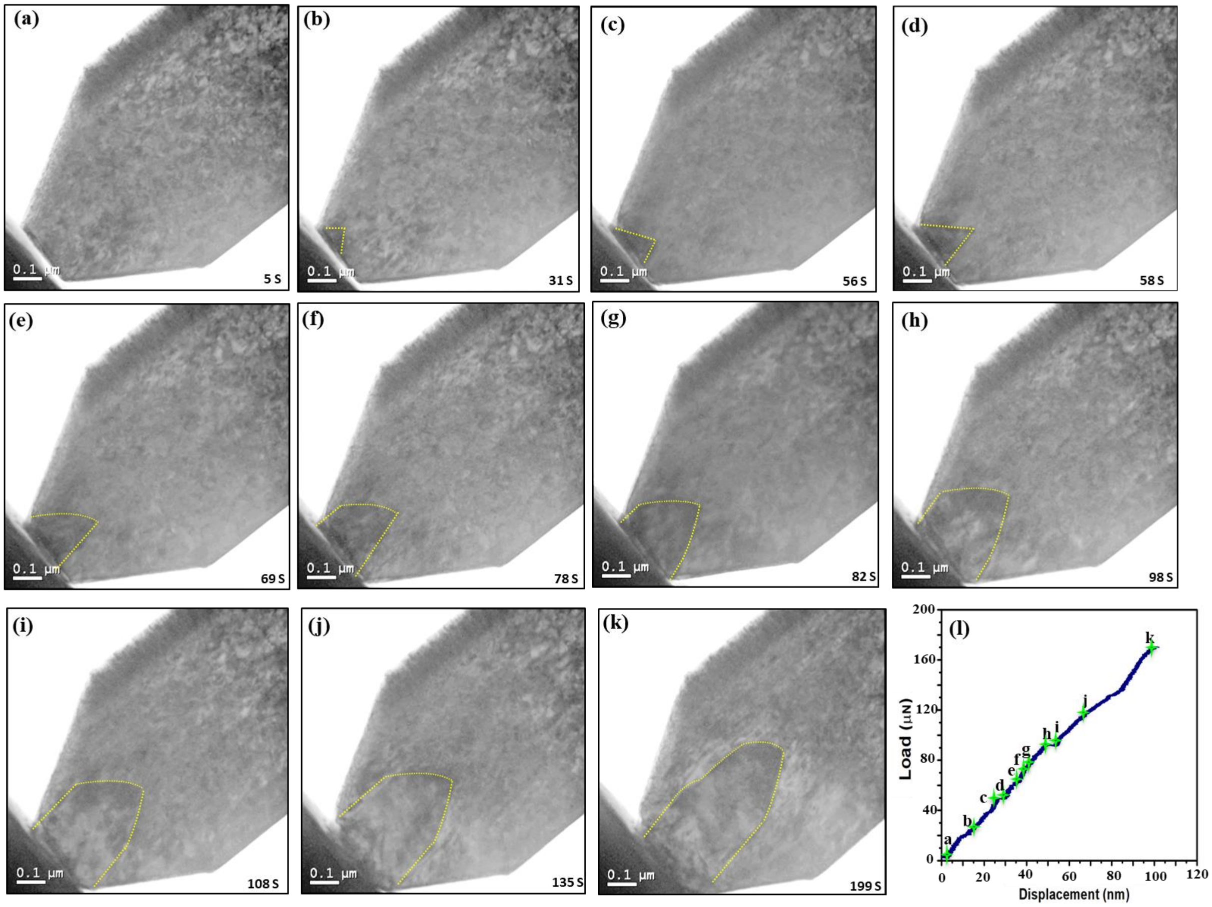

Supplementary Figure 6. Dynamic twinning captured in a wedge-shaped Mg pillar with the top width of 250 nm. (a-k) TEM snapshots showing microstructure evolution, and (l) the in-situ compression load-displacement curve. The dashed lines indicate the twin boundaries. The point b in (l) corresponds to twin nucleation.

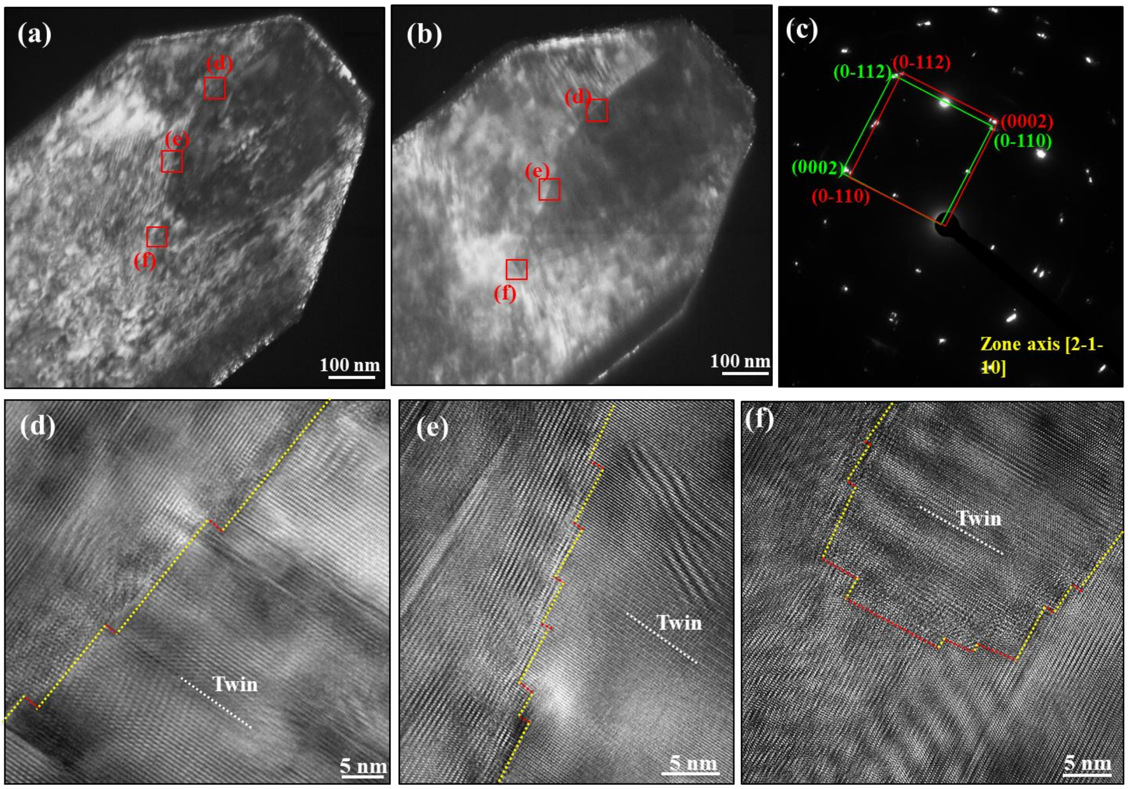

Supplementary Figure 7. The microstructural of the captured twin formed by prismatic-basal transformation in the wedge-shaped pillar with a top width of 250 nm. (a-b) Dark-field TEM image to show the morphology of the twin nucleus, and (c) selected area diffraction pattern to show the orientation relationship between twin and matrix. HRTEM images to show basal/prismatic facets at (d-e) twin boundary or (f) twin tip. The yellow dashed lines indicate the basal-prismatic interfaces and the red lines indicate the prismatic-basal interfaces.

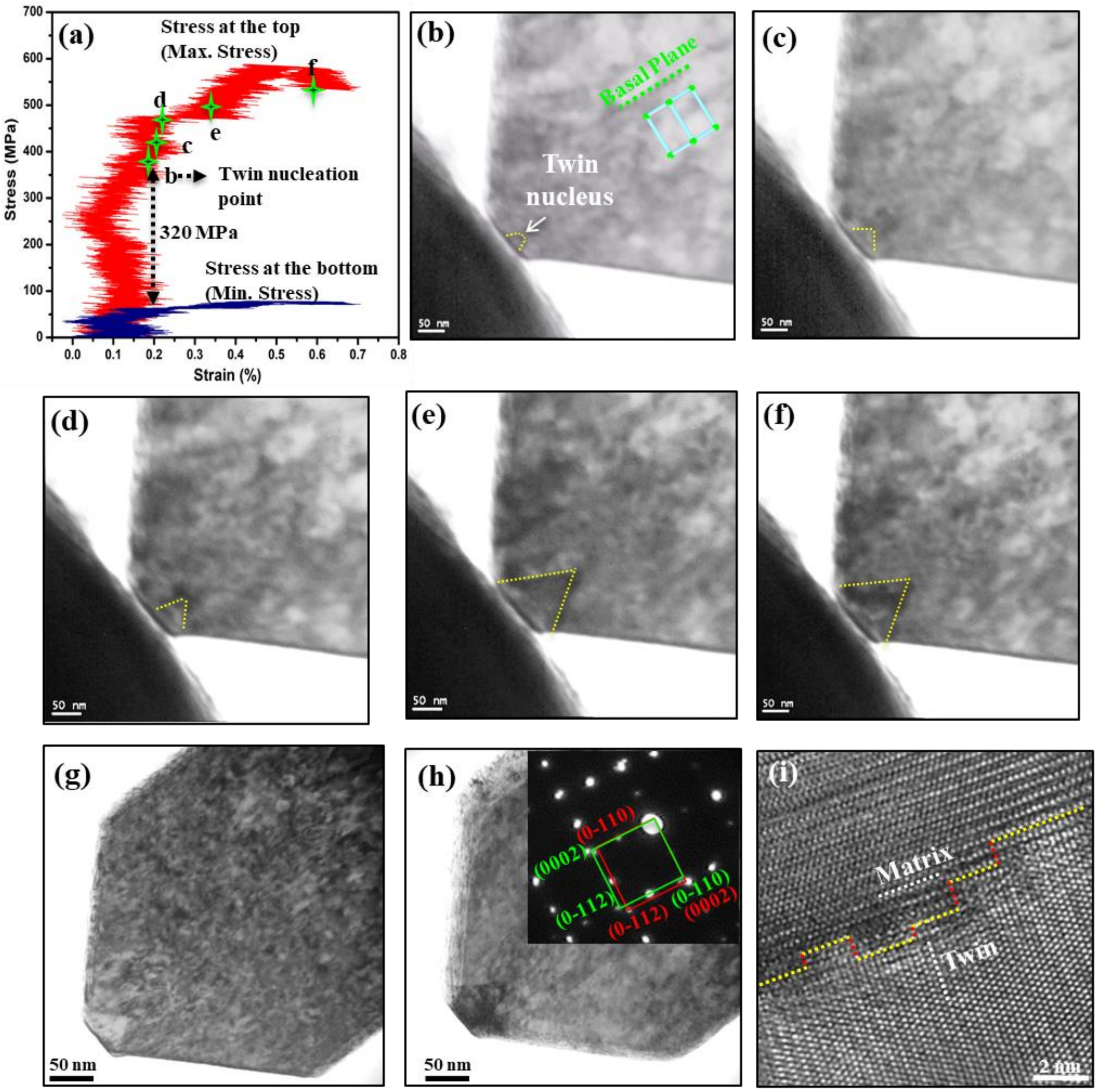

Supplementary Figure 8. Dynamic twinning captured in a wedge-shaped Mg pillar with the top width of 100 nm. (a) corresponding in-situ compression curves of the pillar. The max. stress curve was calculated by the ratios of compression loading to the cross section of the pillar top. The min. stress curve was calculated by the ratios of compression loading to the cross section of the pillar bottom. (b-f) Bright field TEM snap-shots show twin nucleation from the pillar top surface during the in-situ test. (g-i) Bright field TEM images show the morphology of the twin nucleus. The inset of (h) is a selected area diffraction pattern to demonstrate the orientation relationship between twin and matrix. (i) HRTEM images of twin boundaries showing basal/prismatic terraced boundary. The yellow dashed lines indicate the basal-prismatic interfaces, and the red lines indicate the prismatic-basal interfaces.

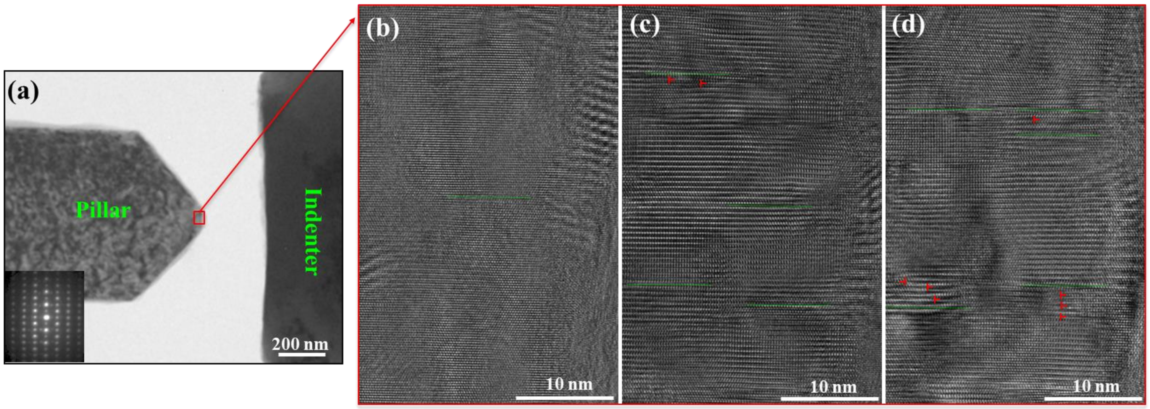

Supplementary Figure 9. In-situ result on a truncated wedge-shaped Mg pillar with a thickness less than 100 nm to show stacking fault and dislocation activity: (a) a bright-field TEM image to show experimental set-up, and the inset is a diffraction pattern for the pillar; (b) atomic resolution TEM images (b) before, (c) during and (d) after the in-situ compression test.

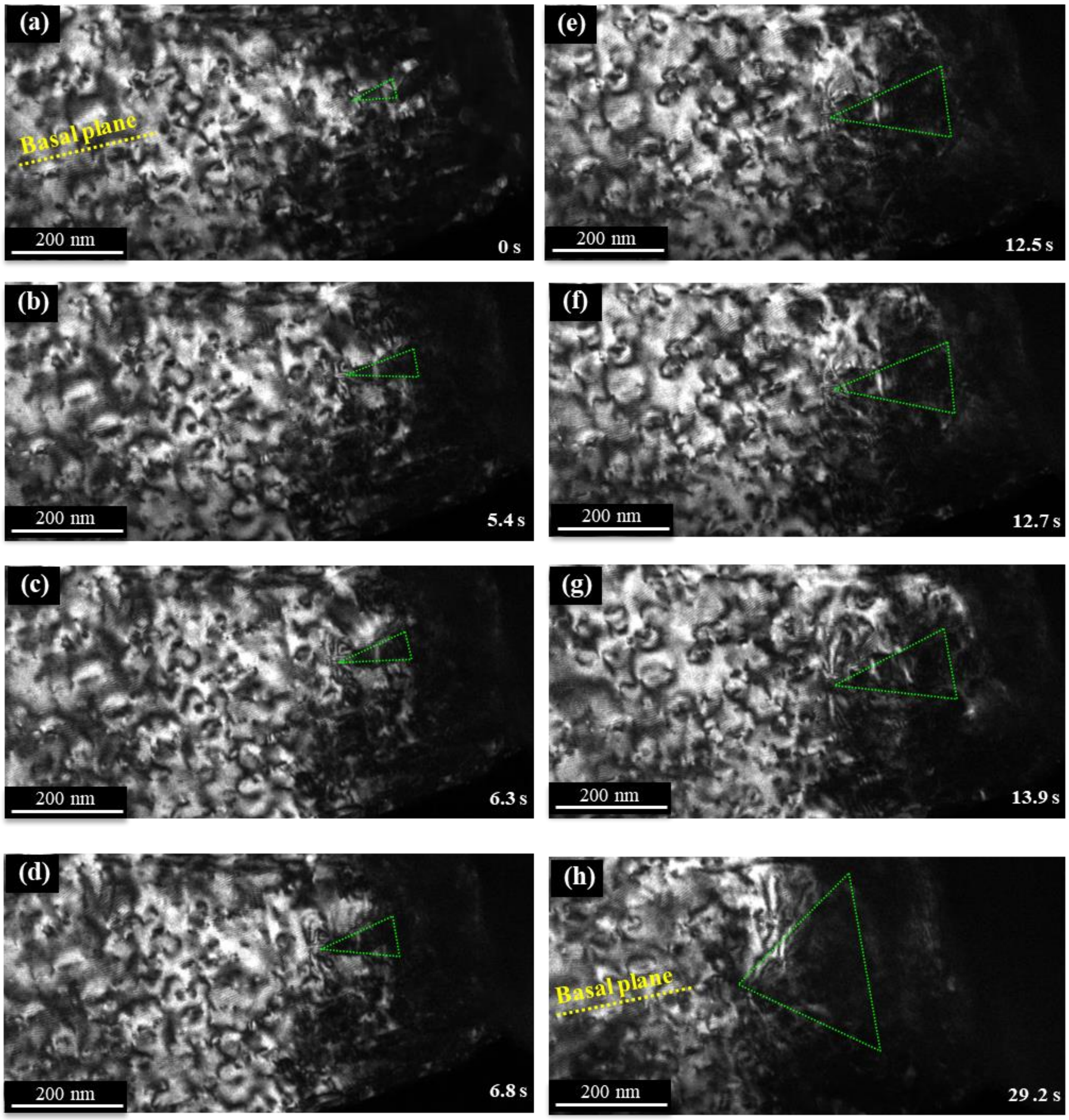

Supplementary Figure 10. In-situ results for a truncated wedge-shaped Mg pillar to show the propagation of a twin along the basal plane: (a-h) dark-field TEM snapshots from the in-situ compression experiment. The corresponding dynamic result is shown in Supplementary Movie 4.

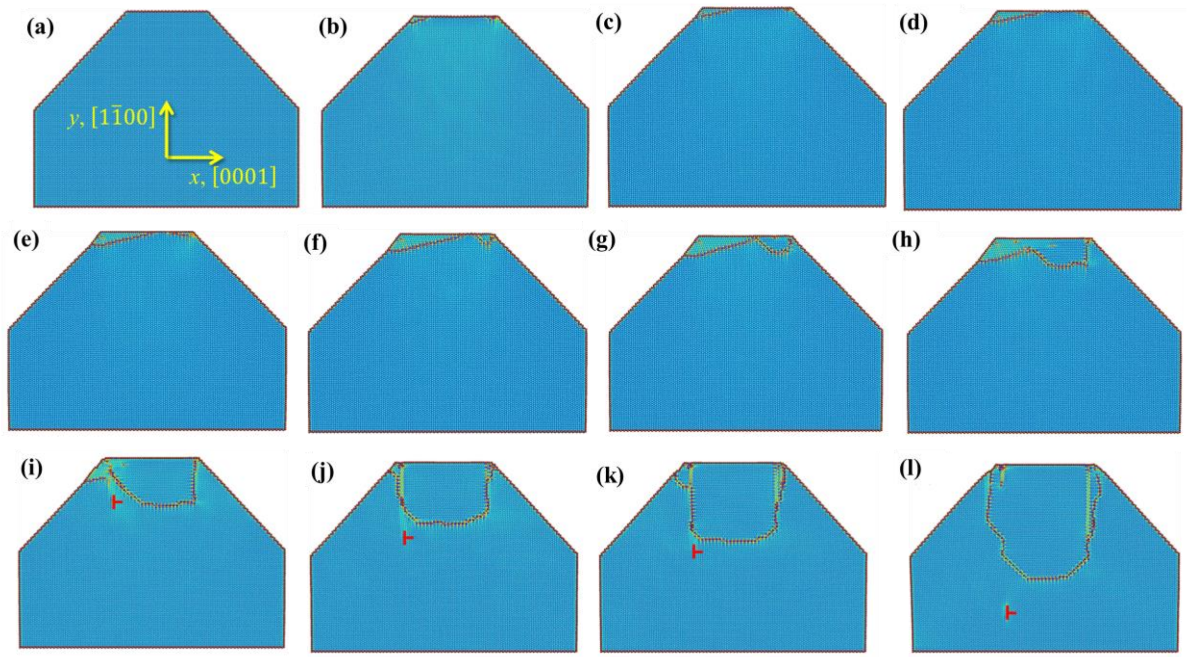

Supplementary Figure 11. Molecular Dynamics simulation of twin nucleation. (a) Initial relaxed single crystal. (b-e) Nucleation of two twin nuclei at the corners on top surface. (f-i) Merge of two twin nuclei into a larger twin nucleus. (j-l) Propagation and growth of twin nucleus via migration of basal-prismatic and prismatic-basal interfaces. The symbol “T” indicates a basal <***a***> dislocation that is emitted from the prismatic-basal boundary. Atoms are color coded according to their excess energy: the lowest in the blue and the highest in the red.

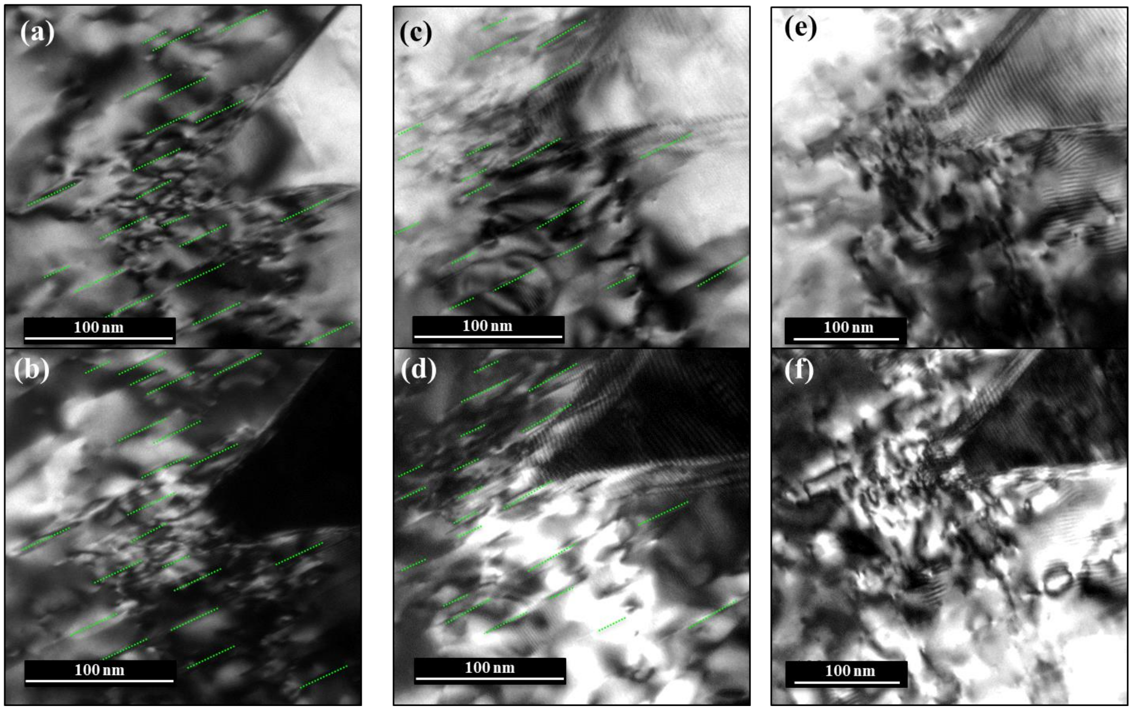

Supplementary Figure 12. Two beam condition TEM images to show dislocations around a twin tip. (a) Bright field TEM, and (b) dark field TEM at g=(01-11) condition; all dislocations are visible at this two beam TEM condition. (c) Bright field TEM, and (d) dark field TEM at g=(01-10) condition. Only <***a***> or <***c***+***a***> dislocations are visible at this two beam TEM condition. (e) Bright field TEM, and (f) dark field TEM at g=(0002) condition. Only <***c***> or <***c***+***a***> dislocations are visible at this two beam TEM condition. The dashed lines indicate locations of the <***a***> dislocations. Most of the dislocations are visible as straight lines at g=(01-10) and g=(01-11) conditions, but not at g=(0002), indicating they are <***a***> dislocations.

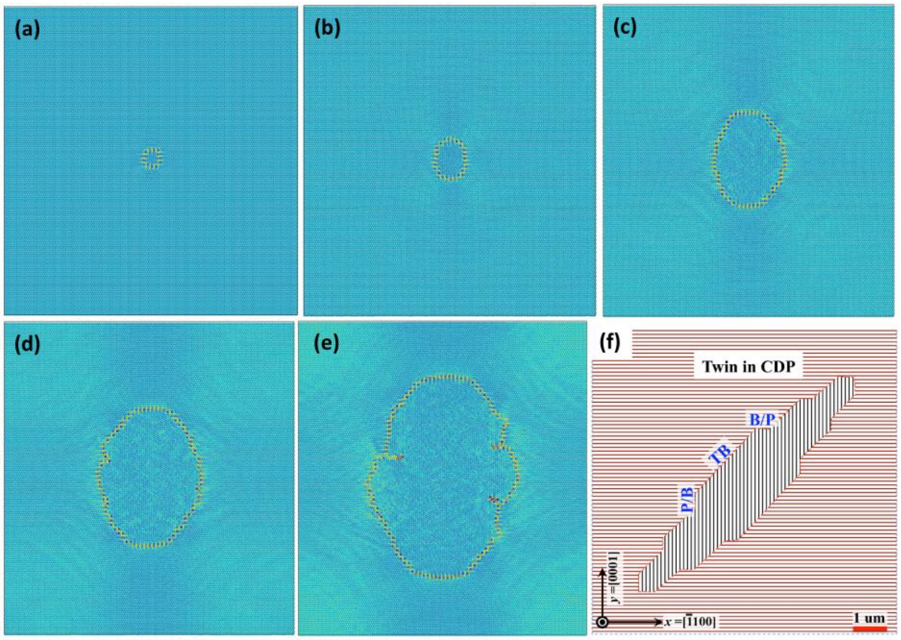

Supplementary Figure 13. Molecular Dynamic simulations of twin growth in a bulk crystal: (a) A 4x4 nm^2 twin nucleus surrounded by prismatic-basal (PB) and basal-prismatic (BP) boundaries is formed in a single crystal. (b-c) Under biaxial loading, tension along the y-direction and compression along the x-direction, the twin nucleus grows initially through the migration of coherent PB and BP boundaries. (d-e) With the growth of the twin, the coherency stress associated with coherent PB and BP interfaces is released in association with the formation of misfit dislocations that further pin the PB and BP interfaces. This leaves the coherent twin bounded by slower growing $\{10\bar{1}2\}$ twin planes. (f) The growing twin then forms the usual lenticular shape with twin boundary terraces. The details can be seen in the Supplementary Movie 7.

Supplementary Tables

Supplementary Table 1 The parameters of rectangular or truncated wedge-shaped pillar (TWP) samples and experimental set-up

Pillar type	Top width	Bottom width	Thickness	Length	Strain speed
Rectangular	750 nm	750 nm	750 nm	1200 nm	0.5 nm/s
TWP	400 nm	750 nm	750 nm	1200 nm	0.5 nm/s
TWP	250 nm	750 nm	750 nm	1200 nm	0.5 nm/s
TWP	100 nm	750 nm	750 nm	1200 nm	0.1 nm/s

Supplementary Discussion

The in-situ compression curves for the regular pillars and TWPs are shown in Supplementary Figure 3. The TEM snapshots in Supplementary Figure 3a-d indicate critical sizes of the twins at the twin nucleation points. The stress data were used to calculate the stress localization levels across the length of the pillars, as shown in Fig. 1i-f. The stress localization levels were calculated by using the max. stress curves subtracting the min. stress curves, as shown in Supplementary Figure 3 i-l. The averaged stress on the cross section of the pillar top is much higher than the averaged stress on the cross section of the pillar bottom, due to the wedge shape of the pillars. At a given pillar bottom width, pillars with shorter top width have larger stress gaps between the top and bottom cross sections, indicating a higher level of stress localization.

In conventional pillar samples, the stress field is uniform (Supplementary Figure 2 a,e,i). Once a twin nucleates, it propagates quickly through the samples. Therefore, it is hard to visualize the initial nucleus; instead one can only observe the “adult” twin and its movement.

There are two proposed early-stage twin growth mechanisms in the conventional pillar: shearing along a twinning plane or prismatic-basal transformation, as schematically shown in Supplementary Figure 4a. First, a narrow $\{10\bar{1}2\}$ twin first initiates at one corner of the pillar and propagates its tip extremely fast across the width of the pillar (see Supplementary Figure 5b and Supplementary Movie 1). The movement of a primary twin boundary and tip indicate its growth mechanism. Since the twin boundary moves mostly along the $(10\bar{1}2)$ twinning plane, this step is likely accomplished by conventional shearing-shuffle associated with successive glide of twinning dislocations [1, 2]. Once its twin tip reaches the other side of the pillars, it then mostly expands in thickness along the basal plane due to the geometric confinement (Supplementary Figure 5c-e).

There are two features worth noting in the conventional pillar. First, twin propagation in the conventional pillar leads to a large strain burst, as seen at point b in Supplementary Figure 5f. Second, and as evidenced by the TEM results (Supplementary Figure 5), the twin grows along the basal plane and ends up with prismatic parallel to basal interfaces (yellow dashed lines in Supplementary Figure 5h) and basal parallel to prismatic interfaces (red dashed lines in Supplementary Figure 5h). This indicates that, in the conventional pillars, twinning can accommodate a large amount of plastic strain via the Prismatic↔Basal transformation.

Using a novel pillar geometry, we introduced a gradient stress field inside a single-crystal pillar to slow down twin propagation. In the TWP with a top width of 400 nm, we observed a two-step early-stage twin growth mechanism, as shown schematically in Supplementary Figure 4b. The first, two twin nuclei initiate from each corner, shear along the twinning plane to meet at the center of the pillar (Supplementary Figure 3b). This step

occurs via the shear-shuffle associated with the gliding of twinning dislocations. Second, once they meet, they stop expanding along the direction of the pillar's width, but mostly both expand along the direction of the pillar's length into the pillar. This occurs via pure-shuffle associated with the basal-prismatic transformation. It is worth nothing that these two twins interacted with each other and thus impede them thickening laterally, which results in stress bursts in the compression curves, as indicated by upward arrows in Supplementary Figure 3j.

In the TWPs with a top width of 250 nm, twinning can be defined as two processes: nucleation and expansion (Figs. S4, S6 and Supplementary Movie 2). A triangular twin can be clearly seen to nucleate from the top surface of the pillar tip (Supplementary Figure 6b). The twin is then found to grow along the direction of pillar's width (Supplementary Figure 6c-f) and length (Supplementary Figure 6g-k). The twinning in this pillar accommodates a more continuous plasticity flow as evidenced by the strain curves (Supplementary Figure 3g,k), which only has a few minor strain bursts. Whereas, in the conventional pillar, the twin propagation results in a much larger strain burst, as shown in Supplementary Figure 3e,i.

Unlike the conventional pillar, the early-stage growth occurs via pure shuffle associated with basal-prismatic transformation, as schematically shown in Supplementary Figure 4. Once the twin nucleates, it mainly propagates its tip and boundary sideways along the prismatic plane and vertically along the basal plane (Supplementary Figure 6 and Supplementary Movie 2). No obvious shearing along the twinning plane was detected. Also, the TEM characterization indicates that the final twin has a bullet-like shape (Supplementary Figure 9a-b). Selected area diffraction pattern (Supplementary Figure 7c)

and HRTEM (Supplementary Figure 7d-f) characterization demonstrate the orientation between matrix and twin. The primary boundaries are basal-prismatic (BP) boundaries in the two sides of the twin (Supplementary Figure 7d). The inclined boundaries are mostly BP terraced interfaces (Supplementary Figure 7e), and the boundary of the twin tip is a prismatic-basal (PB) terraced interface (Supplementary Figure 7f).

In the submicron TWP with the narrowest top width of 100 nm, a small twin nucleus (~20 nm) is revealed to initiate from the top surface (Supplementary Figure 8). The nucleus shows a triangular shape with its boundary and tip parallel to the basal plane, when it was first identified. Postmortem atomic TEM characterization (Supplementary Figure 8h-i) indicates the resulting twin nuclei are both composed of BP and PB terraced boundaries. Therefore, the combination of in-situ and postmortem observation provide evidence that the twin nucleates via the P→B and B→P transformation. In order to clearly identify the early-stage growth event of the twin nucleus, we characterized the twin boundary and tip movement using dark field TEM. As shown in Supplementary Figure 10, once the twin nucleates from the top surface, it moves its tip and boundary along the basal plane (compression direction) under further compression, and no lateral shearing along twin plane is detected. This suggests that crystal geometry confines the twin propagation along the twinning plane and enables the early-stage growth along basal plane via the P→B and B→P transformation.

In order to capture additional atomic-level details of defect activity before twin nucleation, we conducted in-situ tests on the TWPs with a thickness less than 100 nm (Supplementary Figure 9). However, there is a technical challenge on resolving dynamic details at the atomic resolution level during the in-situ compression test, due to un-

avoidable damping issue of the indenter and bending contouring issue of the thin samples. We thus were able to only capture a few snapshots at the atomic resolution level.

In the snapshots (Supplementary Figure 9), we observe dislocation pile-up and stacking faults, which might provide the driving force for the nucleation of twins. However, we failed to produce twins in any pillars with a thickness less than 100 nm. This is possibly due to the "crystal size effect" of the samples [3]. Atomic imaging typically requires a sample thickness no greater than 100 nm. At such a small scale, the stress states on the crystal samples, as well as deformation mechanisms, are different from those in bulk materials, and deformation twinning is generally replaced by ordinary dislocation plasticity and/or local crystal reorientation. Thus, we propose an alternative approach be used to provide support to the proposed "pure shuffle" mechanisms: to characterize twin boundaries and their migration in the thick pillars during the in-situ tests, and then conduct postmortem atomic-resolution TEM characterization on the twin nucleus after the in-situ tests and appropriate thinning of the sample to a thickness appropriate for atomic imaging. At the submicron scale, the stress required for deformation twinning increases with decreasing sample size. This does not change twinning mechanisms in the submicron samples, as twinning was observed to nucleate from pillar surfaces and prismatic/basal interfaces can be identified in all the submicron samples. During the in-situ tests, we identified the BP or PB transformation based on the morphology of the nucleated twins and also the migration direction of the twin boundary. On the one hand, immediately following twin nucleation, the morphology of the twin nucleus indicates how it nucleated. If the twin has a boundary not parallel to the shear direction, but parallel either to a basal or prismatic plane, this indicates that the twin formed from a prismatic/basal transformation. On the other hand,

the migration direction of the twin boundary indicates how a twin propagates. If the twin propagates along either a basal or prismatic plane, instead of the $(10\bar{1}2)$ shearing direction, this suggests that the twin migrates by BP or PB transformation.

There are several empirical potentials for Mg, such as Liu's EAM potential (1996) [4] and Sun's potential (2015) [5]. Recently, one of this paper's authors compared these potentials and density functional theory (DFT) calculations [5] with respect to surface energy, interface energy, twin boundary energy, and formation and migration energies of adatoms, dimers, and trimers on different surfaces. The study shows that the Liu's potential is much closer to the DFT calculations of surface energies and also recreates the dependence of surface energy on normalized atom density, while the Sun potential struggles to recreate the DFT values. Both potentials can reproduce formation energy values for twin boundary and PB interfaces. Thus, atomistic simulations were performed for Mg using empirical interatomic potential developed by Liu *et al.* [4] to explore the twin nucleation process. A TWP is created so that the x-axis is along the $[0001]$ direction, the y-axis is along the $[1\bar{1}00]$ direction, and the z-axis is along the $[11\bar{2}0]$ direction. In Fig. 4 and Supplementary Figure 11, the relaxed TWP has a top width of 12 nm, a bottom width of 40 nm, and a height of 30 nm. Periodic boundary conditions were adopted in the z-direction, while a 1 nm thick region at the bottom of the model is fixed. The periodic depth in the z-direction is 3.0 nm. Due to the periodicity, the produced twin nucleus is eventually straight along the z-direction. An indentation compressive load is applied on the top surface of the model at a displacement rate of 10 m/s at a temperature of 300 K.

Supplementary Figure 11a (0 ps) shows the relaxed wedge-shaped pillar and Supplementary Figure 11b-d (84-90ps) shows the nucleation of twin nuclei at the two

corners of the wedge-shaped model due to the local stress concentrations. Supplementary Figure 11e-i (90-102 ps) shows the merger of two twin nuclei into a larger twin. The propagation and growth of the twin nucleus is shown in Supplementary Figure 11j-l (106-140 ps), which is accomplished by the migration of BP and PB interfaces. During this process, the twin nucleus is mainly surrounded by BP and PB interfaces. Moreover, one basal <*a*> dislocation is emitted from a PB interface. More details can be found in Supplementary Movie 5.

Due to the lattice mismatch, the twin grows via Prismatic↔Basal transformation mechanisms. The high coherency stress can be relaxed through emission of basal dislocations. HRTEM images (Supplementary Figure 12) that were taken from the twin tip clearly show a high density of <***a***> dislocations in the front of twin tip.

We performed MD simulation for initial growth of a twin nucleus that is surrounded by PB and BP boundaries (see Supplementary Figure 13a and Supplementary Movie 6). In the MD simulations, the initial interfaces of a twin consist of (0001) || $(1\bar{1}00)_T$ and $(1\bar{1}00)$|| $(0001)_T$ (referred to as B/P or P/B) when the nucleus is small. The initial nucleus is embedded in a matrix that is subjected to a strain of 0.065 along $[1\bar{1}00]$ and -0.065 along [0001]. MD simulations were carried out at 10 K. Atoms are color coded according to their excess energy: the lowest in the blue and the highest in the red.

When the twin is in a bulk sample that is subjected to effective tensile stress along the <***c***> axis, the resolved shear stress on the basal plane is equal to zero. The emission of <***a***> dislocations from PB and BP interfaces into the matrix is unfavorable, which precludes formation of long PB and BP interfaces. Analogous to the Frank analysis for crystal growth [1], the PB and BP facets grow faster (see Supplementary Figure 13b-c), implying easier

nucleation/propagation of twinning dislocation pairs on PB or BP boundaries than on $\{10\bar{1}2\}$ CTB. With the growth of the twin nucleus, misfit dislocations are nucleated on PB and BP boundaries to release the coherency stress and further pin the PB and BP interfaces (see Supplementary Figure 13d-e). This leaves the coherent twin bounded by slower growing $\{10\bar{1}2\}$ twin planes. The growing twin then forms the usual lenticular shape, Supplementary Figure 13f, with CTB terraces. The shape formed in this case is consistent with the twin morphology found in bulk samples [1].

Supplementary References

1. Gong M, Hirth JP, Liu Y, Shen Y, Wang J. Interface structures and twinning mechanisms of twins in hexagonal metals. *Materials Research Letters* **5**, 449-464 (2017).

2. Wang J, Yadav SK, Hirth JP, Tomé CN, Beyerlein IJ. Pure-Shuffle Nucleation of Deformation Twins in Hexagonal-Close-Packed Metals. *Materials Research Letters* **1**, 126-132 (2013).

3. Yu Q, *et al.* Strong crystal size effect on deformation twinning. *Nature* **463**, 335-338 (2010).

4. Xiang-Yang L, James BA, Furio E, John AM. EAM potential for magnesium from quantum mechanical forces. *Modelling and Simulation in Materials Science and Engineering* **4**, 293 (1996).

5. Chu H, Huang H, Wang J. Clustering on Magnesium Surfaces – Formation and Diffusion Energies. *Scientific Reports* **7**, 5167 (2017).
