# Supplementary material for: Visualization and validation of twin nucleation and early-stage growth in magnesium
Source: Nat Commun. 2022 Jan 10;13:20. doi: 10.1038/s41467-021-27591-z (PMC8748725; doi:10.1038/s41467-021-27591-z)
Supplement: Supplementary file 3 — Description of Additional Supplementary Files [file 41467_2021_27591_MOESM3_ESM.pdf]

Description of Additional Supplementary Files

File Name: Supplementary Movie 1
Description: In-situ video to show twinning behavior in the conventional pillar with width of 750 nm and thickness of 750 nm. The video was accelerated by 8 times.

File Name: Supplementary Movie 2
Description: In-situ video to show twinning behavior in the wedge-shaped pillar with top width of 250 nm and thickness of 750 nm. The video was accelerated by 8 times.

File Name: Supplementary Movie 3
Description: In-situ video to show twinning behavior in the wedge-shaped pillar with top width of 100 nm and thickness of 750 nm. The video was accelerated by 8 times.

File Name: Supplementary Movie 4
Description: In-situ dark field TEM video of a truncated wedge-shaped Mg pillar to show the early-stage growth of a twin tip along the basal plane.

File Name: Supplementary Movie 5
Description: Molecular dynamic simulation video to show twin nucleation via prismatic-basal transformation from the top surface of the wedge-shaped pillar.

File Name: Supplementary Movie 6
Description: Molecular dynamic simulation of twin growth in a bulk single crystal.
